# Association Between Obesity and Post-stroke Anxiety in Patients With Acute Ischemic Stroke

**DOI:** 10.3389/fnut.2021.749958

**Published:** 2021-11-25

**Authors:** Bei-Lei Zhu, Ai-Yi Hu, Gui-Qian Huang, Hui-Hua Qiu, Xian-Chai Hong, Ping-Lang Hu, Cheng-Xiang Yuan, Yi-Ting Ruan, Bo Yang, Jin-Cai He

**Affiliations:** ^1^Department of Neurology, The First Affiliated Hospital of Wenzhou Medical University, Wenzhou, China; ^2^Department of First Clinical Medical School, The First Affiliated Hospital of Wenzhou Medical University, Wenzhou, China; ^3^School of Public Health and Management, Wenzhou Medical University, Wenzhou, China

**Keywords:** post-stroke anxiety, obesity, abdominal obesity, stroke, anxiety

## Abstract

Post-stroke anxiety (PSA) is serious psychosomatic comorbidity among patients with stroke, but whether obesity could be positively associated with PSA is currently unknown. The purpose of this study was to investigate the potential association between obesity and subsequent anxiety risk in patients with stroke. A total of 441 patients with acute ischemic stroke (AIS) onset were consecutively recruited within 7 days, and PSA and post-stroke depression (PSD) were evaluated by using a 14-item Hamilton anxiety scale (HAMA) and 17-item Hamilton depression scale (HAMD) at the end of 1-month follow-up. The odds ratio (OR) with 95% CI was estimated for the incidental PSA by using logistic regression analysis. The incidence of PSA was 25.85% at the end of 1-month follow-up, with a significant difference between patients with and without abdominal obesity. Relative fat mass (RFM) and abdominal obesity were significantly associated with an elevated risk of PSA, and the crude ORs were 1.04 (95% CI: 1.01–1.08) and 1.93 (95% CI: 1.11–3.34), respectively. Even after adjustment for obesity-related risk factors and PSA-related clinical measurements, the association remained to be pronounced with abdominal obesity. However, RFM (OR = 1.03, 95% CI: 0.99–1.06, *P* = 0.053) and abdominal obesity (OR = 1.31, 95% CI: 0.80–2.15, *P* = 0.280) were not significantly associated with an elevated risk of PSD. Abdominal obesity was independently associated with the PSA instead of PSD, which may help predict PSA risk in clinical practice. Further prospective clinical studies with a long follow-up duration are warranted to verify this finding.

## Introduction

Stroke is a major cause of long-term disability in elderly people, and stroke-related high morbidity, disability, and mortality have become a global public health problem in the world (1). Post-stroke anxiety (PSA) is a common and serious psychosomatic comorbidity among patients with stroke (2). A previous study showed that anxiety disorders diagnosed occurred in ~20–25% of patients during any time of onset stroke (3). Several studies also showed that 21% of patients with stroke suffered from moderate or severe anxiety when stroke in 3 months after a stroke (4), and the frequency of PSA was 18% at 2 years (5). The prognosis of PSA is poor and PSA interferes substantially with social life and functional recovery (6). Therefore, early identification of PSA is of great significance to improve the stroke prognosis.

A meta-analysis of 25 studies revealed that anxiety occurred more frequently in obese/overweight than in patients with normal weight (7), and there was a positive association between obesity and anxiety disorders (8). Abdominal obesity, also known as central obesity, refers to the abnormal accumulation of adipose tissue in the abdominal cavity or around the abdomen. Waist circumference (WC) is widely used in the evaluation of abdominal obesity due to a few advantages in easy assessments, low cost, and high correlations with visceral fat savings (9). According to Chinese adult weight determinations (WS/T 428-2013), abdominal obesity was defined as WC ≥90 cm for men and ≥85 cm for women (10). In addition, relative fat mass (RFM) is a simple linear equation based on the height-to-waist ratio, as a potential alternative tool to estimate whole-body fat percentage in women and men 20 years of age and older, which was proposed in 2018(11). The data showed higher sensitivity (lower false negatives) of RFM for body fat-defined obesity among women and men, supporting the potential of RFM as a screening tool for obesity (11).

A review found good evidence that obesity is prospectively associated with increased anxiety, depression, and other emotional regulation disorders (12). Among the people with anxiety and depression, 50% of women and 41% of men are obese (13). Whether the association between obesity and anxiety would occur among patients with stroke is currently unknown. The purpose of this study was to investigate the potential association between obesity and subsequent anxiety risk in patients with stroke.

## Materials and Methods

### Study Participants

The patients with acute stroke were consecutively admitted to the Stroke Unit of the First Affiliated Hospital of Wenzhou Medical University in the present study from October 2013 to September 2014. Inclusion criteria include (1) incident stroke within 7 days, (2) 18–80 years old, and (3) the informed consent that the patients have signed. Exclusion criteria include (1) inability to complete the neuropsychological test due to dementia, severe dysarthria, severe aphasia, and deafness after stroke, (2) previous history of anxiety, depression, or mental illness, (3) previous brain trauma, Parkinson's disease, and other organic brain lesions, (4) patients with fatty liver and metabolic disease, and (5) patients with other serious medical conditions that prevented follow-up studies. This was determined by interviewing patients and examining medical records. A total of 469 patients were included in the study, of which, 28 patients cannot be contacted by telephone 1 month postdischarge and were considered lost to follow-up. Thus, the final study population enrolled 441 patients. This study followed ethical guidelines and obtained the approval of the Institutional Review Board of the First Affiliated Hospital of Wenzhou Medical University.

### Clinical Measurements

The data of this study were collected *via* standardized questionnaires within 24 h after admission, which included demographic characteristics (age, marital status, education, etc.), lifestyle characteristics (smoking status, alcohol intake, etc.), health status, and medical history, conducted in face-to-face interviews by trained physicians. The National Institutes of Health Stroke Scale (NIHSS) was used to evaluate the stroke severity on admission. NIHSS is a neurologic examination scale including 15 items. The higher the score is, the more serious the neurologic impairment is. The Barthel Index (BI) was used for the evaluation of activities of daily living (ADL). The lower the score is, the worse the ability of daily living. Fasting blood samples were obtained from all patients within 24 h after admission.

The neurological physicians used the Hamilton anxiety scale (HAMA) with 14 items and the Hamilton depression scale (HAMD) with 17 items to assess anxiety and depression symptoms during the 1-month follow-up (14, 15). Patients with a HAMA anxiety score >7 were considered to have existing symptoms of anxiety and those with a HAMD depression score >7 were considered to have existing symptoms of depression according to the structured clinical interview of the diagnostic and statistical manual of mental disorders, 4th edition (14, 15). The assessment was done by two independent senior neuropsychologists who were blinded to clinical characteristics. They had both undergone professional training in neuropsychological assessment and obtained assessment qualifications to ensure the consistency of the assessment results.

### Obesity and Obesity-Related Indicator Measurements

The following indicators were collected within 24 h of admission. Physical indicators: include height, weight, and WC. Both height and weight were measured according to the standard anthropometric method, and WC is the length of circumference measured at the horizontal position of the middle point of the line between the inferior margin of the costal arch of the axillary midline and iliac spine when breathing quietly (10). The above data were used to calculate the body mass index (BMI) and RFM of each patient. BMI = weight (kg)/ height^2^ (m^2^). According to WS/T 428-2013 adult weight measurement, BMI ≥ 28 kg/m^2^ is defined as obesity and abdominal obesity was defined as WC ≥90 cm for men and ≥85 cm for women (10). RFM = 64–[20 × (height/WC) + (12 × sex)]; sex = 0 for men and 1 for women (11).

### Statistical Analyses

The results were expressed as percentages for categorical variables, median (interquartile range, IQR) for continuous variables with non-normal distribution, and mean (SD) for continuous variables with normal distribution. The normality of data was evaluated using Kolmogorov-Smirnov test and P-P plots. The classification variables were analyzed by the Chi-square test. The continuous variables of non-normal distribution were analyzed by Mann-Whitney U-test and Kruskal-Wallis test. The continuous variables of normal distribution were analyzed by ANOVA. The univariate and multivariate logistic regression was used to analyze the predictors of PSA at 1 month. In order to control the influence of confounding factors, variables with statistical significance in the univariate logistic regression analysis were taken as independent variables and were respectively introduced into the multivariate logistic regression model with PSA as the dependent variable. The receiver operating characteristic (ROC) curve was used to compare the predictive value of different indicators for PSA. *p* < 0.05 were considered as statistically significant. MedCalc 18.2.1 was used to compare the area under the curve of different indexes. All data were performed by the IBM Statistical Product and Service Solutions (SPSS) Statistics (version 20.0) and MedCalc 18.2.1.

## Results

### Baseline Characteristics

**Figure 1:**
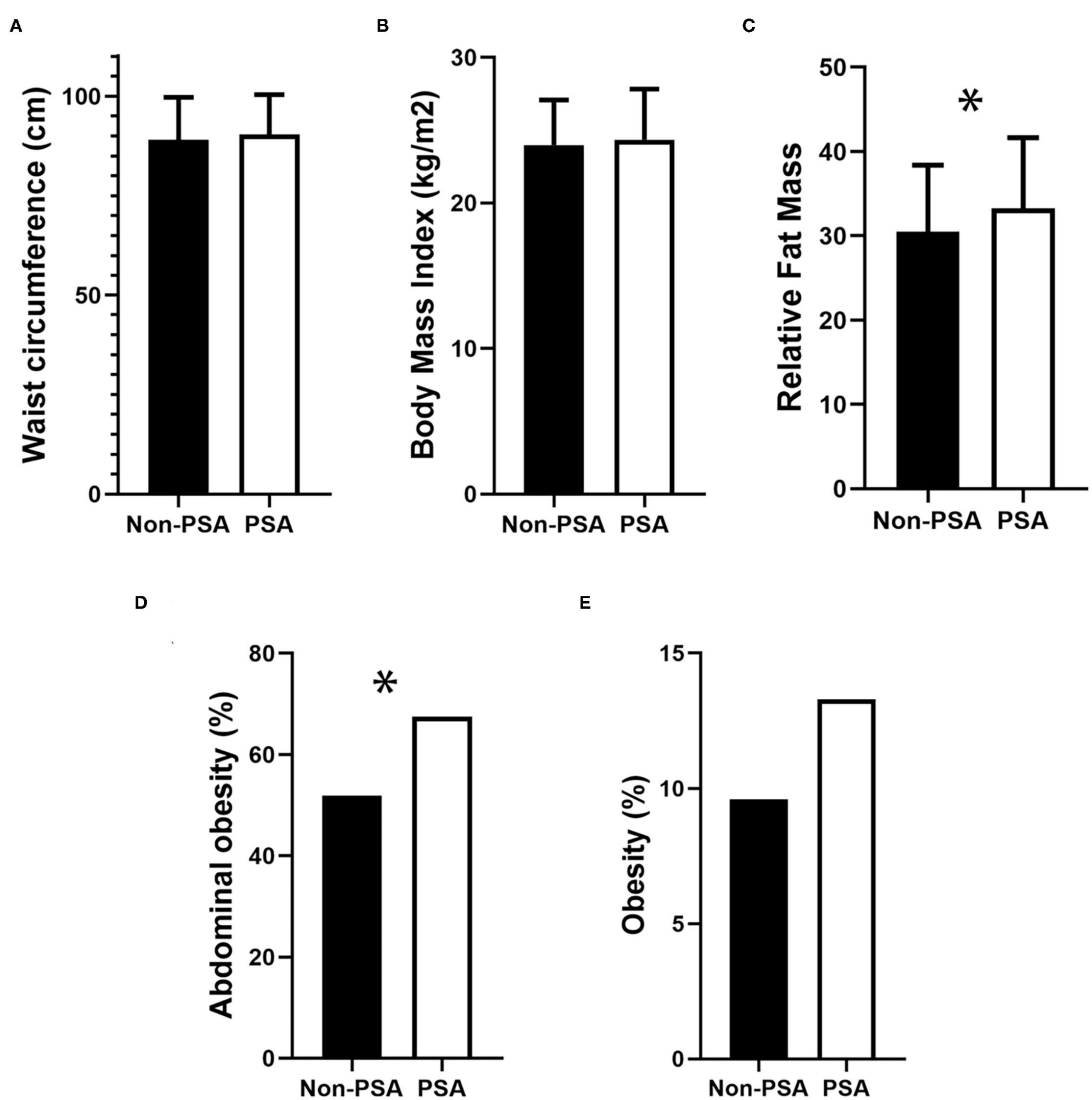
Comparison of obesity-related indicators in PSA and Non-PSA group. **(A)** Waist circumference, **(B)** Body Mass Index, **(C)** Relative Fat Mass, **(D)** Abdominal obesity, and **(E)** Obesity. *p < 0.05.

A total of 441 patients with an average age of 62.33 years were included in this study, and 63.8% were male. In the total participants, the incidence of PSA and post-stroke depression (PSD) was 25.85% (95% CI: 21.98–30.13%) and 35.60% (95% CI: 31.27–40.18%) at the end of 1-month follow-up, respectively. The differences in background characteristics and clinical indicators between the PSA group and the non-PSA group were shown in Table 1. The differences in background characteristics and clinical indicators between the PSD group and the non-PSD group were shown in Table 2. We found significant differences between patients with PSA and non-PSA in NIHSS score (*P* = 0.003), BI score (*P* < 0.001), fasting plasma glucose (*P* = 0.024), height (*P* = 0.010), RFM (*P* = 0.012), and abdominal obesity (*P* = 0.018). Figure 1 presents the comparisons of obesity indicators between the PSA and non-PSA groups. The PSA group tend to have a higher level of WC (90.36 ± 10.02 vs. 89.00 ± 10.73) and BMI (24.36 ± 3.47 vs. 23.99 ± 3.10) as well as a higher incidence of obesity (13.3 vs. 9.6%) than the patients with non-PSA, but the differences were not statistically significant (*P* = 0.331, 0.303, and 0.279, respectively). No significant differences between patients with PSA and non-PSA were found in demographic characteristics or stroke risk factors. We also found significant differences between patients with PSD and non-PSD in NIHSS score (*P* < 0.001), BI score (*P* < 0.001), height (*P* = 0.009), and no significant differences in demographic characteristics, stroke risk factors, or other obesity-related indicators.

**Table 1:** Baseline demographic and clinical characteristics between patients with PSA and non-PSA.

**Characteristics**	**Non-PSA (*n* = 327)**	**PSA (*n* = 114)**	***p*-value**
**Demographic characteristics**			
Age, mean ± SD (year)	62.55 ± 10.26	63.3 ± 10.53	0.824
Male, *n* (%)	216 (66.5%)	63 (56.2%)	0.052
Education, median (IQR)	4 (0-7)	3 (0-6)	0.166
Religion, *n* (%)	156 (48.4%)	57 (52.3%)	0.488
Married, *n* (%)	301 (92.0%)	98 (86.0%)	0.295
**Stroke risk factors**			
History of hypertension, *n* (%)	234 (72.2%)	84 (74.3%)	0.664
History of diabetes, *n* (%)	70 (21.7%)	29 (25.7%)	0.383
Coronary artery disease, *n* (%)	24 (7.5%)	6 (5.4%)	0.443
History of hyperlipidemia, *n* (%)	27 (8.4%)	13 (11.5%)	0.324
History of a previous stroke, *n* (%)	31 (9.6%)	11 (9.7%)	0.966
Current smoking, *n* (%)	105 (32.6%)	30 (27.0%)	0.425
Current drinking, *n* (%)	119 (38.4%)	36 (33.3%)	0.494
**Neurologic function**			
NIHSS score, median (IQR)	2 (1-4)	3 (1-6)	0.003
BI score, median (IQR)	90 (60-100)	75 (45-95)	<0.001
**Laboratory parameters**			
Fasting plasma glucose, mean ± SD (mmol/L)	5.59 ± 1.95	6.11 ± 2.38	0.024
Cholesterol, mean ± SD (mmol/L)	4.77 ± 1.16	4.85 ± 1.20	0.531
Triglyceride, mean ± SD (mmol/L)	1.80 ± 1.18	1.65 ± 0.94	0.239
HDL, mean ± SD (mmol/L)	1.09 ± 0.31	1.12 ± 0.35	0.517
LDL, mean ± SD (mmol/L)	2.78 ± 0.98	2.96 ± 0.95	0.100
**Obesity-related measurements**			
Height, mean ± SD (cm)	164.59 ± 7.37	162.4 ± 7.38	0.010
Weight, mean ± SD (kg)	65.11 ± 10.09	64.3 ± 10.89	0.493
WC, mean ± SD (cm)	89.00 ± 10.73	90.36 ± 10.02	0.331
BMI, mean ± SD (kg/m^2^)	23.99 ± 3.10	24.36 ± 3.47	0.303
RFM, mean ± SD	30.51 ± 7.87	33.24 ± 8.41	0.012
Obesity, *n* (%)	30 (9.6%)	14 (13.3%)	0.279
Abdominal obesity, *n* (%)	109 (51.9%)	52 (67.5%)	0.018

*BI, modified Barthel index; BMI, body mass index; IQR, interquartile range; NIHSS, National Institutes of Health Stroke Scale; PSA, post-stroke anxiety; RFM, relative fat mass; WC, waist circumference*.

**Table 2:** Baseline demographic and clinical characteristics between PSD and non-PSD patients.

**Characteristics**	**Non-PSD (*n* = 284)**	**PSD (*n* = 157)**	***p*-value**
**Demographic characteristics**			
Age, mean ± SD (year)	62.37 ± 10.19	62.70 ± 10.58	0.750
Male, *n* (%)	190 (67.1%)	89 (57.8%)	0.052
Education, median (IQR)	4 (0-7)	4 (0-6)	0.378
Religion, *n* (%)	141 (50.9%)	72 (47.4%)	0.529
Married, *n* (%)	259 (91.2%)	140 (89.2%)	0.842
**Stroke risk factors**			
History of hypertension, *n* (%)	205 (72.7%)	113 (72.9%)	0.963
History of diabetes, *n* (%)	59 (21.0%)	40 (25.8%)	0.251
Coronary artery disease, *n* (%)	22 (7.9%)	8 (5.2%)	0.287
History of hyperlipidemia, *n* (%)	22 (7.9%)	18 (11.6%)	0.194
History of a previous stroke, *n* (%)	24 (8.5%)	18 (11.6%)	0.298
Current smoking, *n* (%)	92 (32.7%)	43 (28.3%)	0.542
Current drinking, *n* (%)	112 (41.2%)	43 (29.5%)	0.148
**Neurologic function**			
NIHSS score, median (IQR)	2 (1–4)	3.5 (2–6)	<0.001
BI score, median (IQR)	95 (70–100)	62.5 (40–90)	<0.001
**Laboratory parameters**			
Fasting plasma glucose, mean ± SD (mmol/L)	5.62 ± 1.98	5.92 ± 2.25	0.159
Cholesterol, mean ± SD (mmol/L)	4.75 ± 1.14	4.81 ± 1.16	0.635
Triglyceride, mean ± SD (mmol/L)	1.81 ± 1.14	1.69 ± 1.01	0.317
HDL, mean ± SD (mmol/L)	1.14 ± 0.29	1.10 ± 0.31	0.208
LDL, mean ± SD (mmol/L)	2.78 ± 0.94	2.90 ± 0.95	0.209
**Obesity-related measurements**			
Height, mean ±SD (cm)	164.74 ± 7.25	162.75 ± 7.58	0.009
Weight, mean ± SD (kg)	65.11 ± 10.09	64.3 ± 10.89	0.493
WC, mean ± SD (cm)	23.93 ± 3.08	24.36 ± 3.41	0.188
BMI, mean ± SD (kg/m2)	89.27 ± 10.35	89.53 ± 10.94	0.840
RFM, mean ± SD	30.55 ± 7.99	32.48 ± 8.18	0.056
Obesity, *n* (%)	100 (53.8%)	61 (60.4%)	0.280
Abdominal obesity, *n* (%)	25 (9.2%)	19 (13.1%)	0.211

*BI, modified Barthel index; BMI, body mass index; IQR, interquartile range; NIHSS, National Institutes of Health Stroke Scale; PSD, post-stroke depression; RFM, relative fat mass; WC, waist circumference*.

### Logistic Analyses

The associations of obesity and obesity-related measurements with PSA were showed in Table 3. The univariate logistic regression analysis found that the RFM (OR = 1.04; 95% CI: 1.01–1.08; *P* = 0.013), abdominal obesity (OR = 1.93; 95% CI: 1.11–3.34; *P* = 0.019), fasting blood glucose level (OR = 1.12; 95% CI: 1.01–1.23; *P* = 0.027), height (OR = 0.96; 95% CI: 0.93–0.99; *P* = 0.011), NIHSS score (OR = 1.13; 95% CI: 1.05–1.22; *P* = 0.001), and BI score (OR = 0.98; 95% CI: 0.97–0.99; *P* < 0.001) to be significantly associated with PSA risk, but this association remained to be pronounced with abdominal obesity (OR = 2.15; 95% CI: 1.01–4.60; *P* = 0.047) and fasting plasma glucose (OR = 1.15; 95% CI: 1.01–1.30; *P* = 0.030) in multivariate-adjusted logistic analyses. Other obesity-related measurements were not significantly associated with PSA.

**Table 3:** Univariate and multivariate logistic models of clinical determinants of PSA.

**Variables**	**Univariate analysis**	***p*-value**	**Multivariate analysis^a^**	***p*-value**
	**OR (95% CI)**		**OR (95% CI)**	
RFM	1.04 (1.01–1.08)	0.013	0.99 (0.94–1.05)	0.809
Abdominal obesity	1.93 (1.11–3.34)	0.019	2.15 (1.01–4.60)	0.047
Obesity	1.45 (0.74–2.86)	0.281		
Fasting blood glucose	1.12 (1.01–1.23)	0.027	1.15 (1.01–1.30)	0.030
Height	0.96 (0.93–0.99)	0.011	0.95 (0.89–1.00)	0.058
NIHSS score	1.13 (1.05–1.22)	0.001	1.06 (0.95–1.19)	0.281
BI score	0.98 (0.97–0.99)	<0.001	0.99 (0.98–1.00)	0.118

a *Adjusted for RFM, fasting blood glucose level, height, NIHSS score, and BI score*.

*BI, modified Barthel index; NIHSS, National Institutes of Health Stroke Scale; OR, odds ratio; RFM, relative fat mass*.

### ROC Analyses

**Figure 2:**
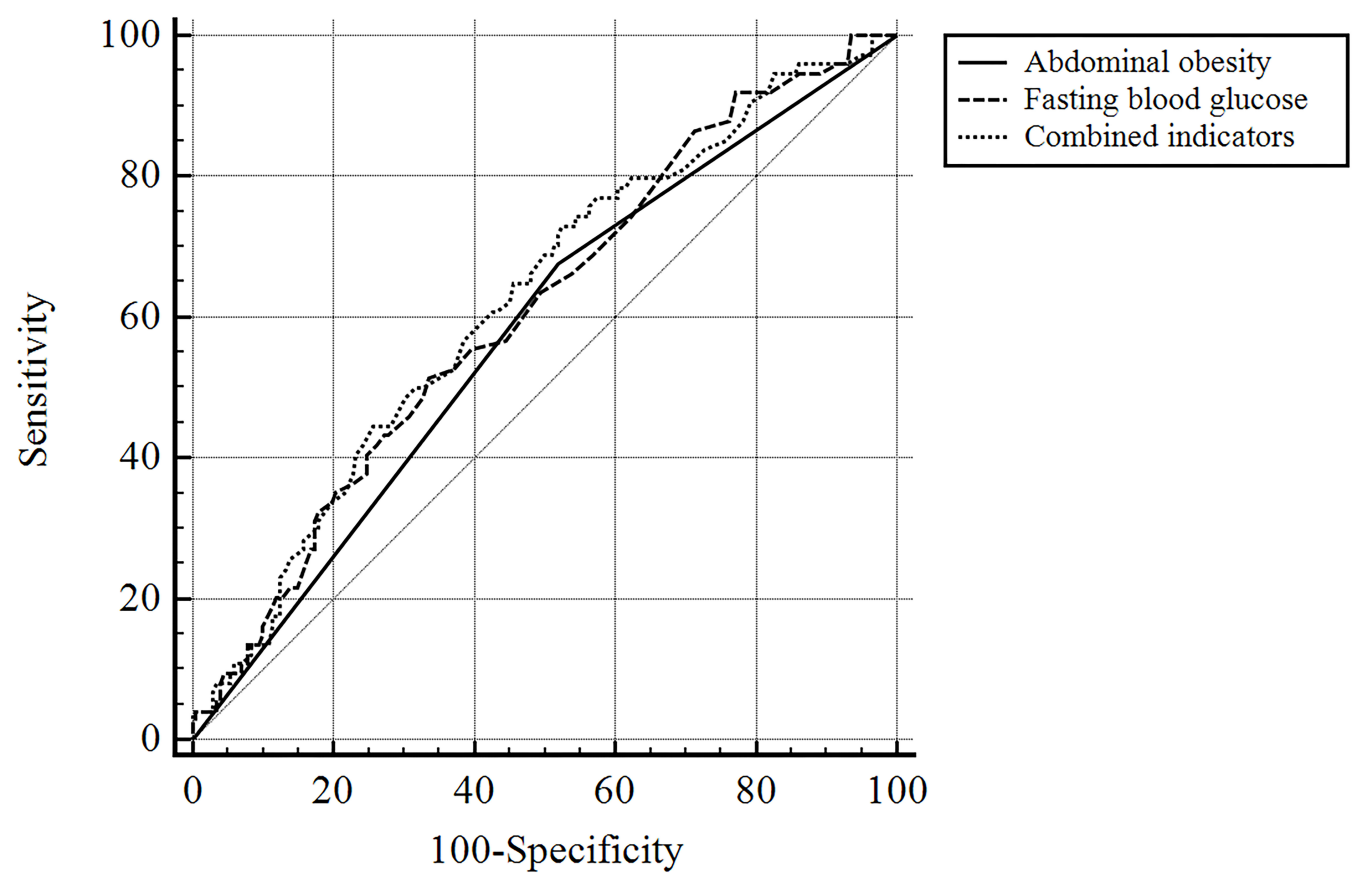
The ROC curve of predictive value of abdominal obesity, fasting blood glucose and their integrated model for PSA.

The ROC curve and the area under the curve (AUC) were used to further compare the predictive value between abdominal obesity, fasting blood glucose, and their integrated model (Figure 2; Table 3). The areas under the ROC curve of abdominal obesity alone, fasting blood glucose alone, and combination of them for PSA were 0.578 (95% CI: 0.517–0.637; *P* = 0.047), 0.608 (95% CI: 0.548–0.666; *P* = 0.006), and 0.622 (95% CI: 0.562–0.670; *P* = 0.002), respectively. The optimal cutoff for fasting blood glucose was 5.45 mmol/L, at which the sensitivity of 51.4% and specificity of 66.3% were observed. The index of abdominal obesity combined with fasting blood glucose had a larger AUC area than the single index of abdominal obesity (abdominal obesity combined with fasting blood glucose vs. abdominal obesity, *P* = 0.05; abdominal obesity combined with fasting blood glucose vs. fasting blood glucose, *P* = 0.65; abdominal obesity *vs*. fasting blood glucose, *P* = 0.50).

## Discussions

As we all know, PSA has many hazards. There are many risk factors associated with PSA, including depression (16), cognitive impairment (17), lesion location (18), and fatigue (19). Obesity may also be a risk factor.

However, few studies have reported the association between obesity and PSA, and our study confirmed the correlation between obesity and PSA to some extent. Abdominal obesity was significantly associated with PSA in both univariate and multivariate analyses, indicating abdominal obesity defined by WC is an independent risk factor for PSA. Previous studies also confirmed that the increased WC is associated with depression and anxiety tendency in both genders, which is consistent with our results (20).

Anxiety is one of the most common mental disorders in obese patients (21). Laboratory data have suggested anxiety-like behaviors may have increased in rodents that are genetically predisposed to obesity, e.g., *db/db* mice, and in high-fat diet (HFD)-induced obesity (22–24), which may be biologically related to inflammation and altered hormone signaling. Obese patients are accompanied by increased levels of interleukin and other inflammatory factors, which may significantly influence neurotransmission in regulating brain circuits, thereby causing emotional responses (25). In addition, it is known that hyperactivity of the hypothalamus pituitary adrenal (HPA) axis can lead to visceral fat accumulation and abdominal obesity, which has been considered as one of the basic biological mechanisms underlying the emotional disorder (26, 27). As the feedback inhibition of glucocorticoid (GC) on the HPA axis signal decreased, the levels of the cytoplasmic glucocorticoid receptor (GR) in the prefrontal cortex (PFC) and hippocampus were significantly changed, which were involved in the pathogenesis of emotional disorder (25). Emotional disorders include anxiety and depression, but more specific pathophysiological links between obesity and anxiety remain unclear. Anxiety-like behavior in obesity may be caused by an increased burden on senescent cells (28). Clearing senescent cells from high fat-fed or leptin receptor-deficient obese mice restored neurogenesis and alleviated anxiety-related behavior (28).

We also found that the indicators of obesity, including WC, BMI, and obesity incidence, did not reach statistical significance between the patients with PSA and the patients with non-PSA. A possible explanation was that BMI may be a low sensitivity index to evaluate the incident obesity. As we mentioned earlier, the relationship between BMI and anxiety remains controversial. A recent study found that BMI was significantly higher among overweight/obese patients who had anxiety or depression, and anxiety and depression scores were positively correlated with BMI (29). Similarly, a study shows that a U-shaped association between anxiety and BMI after adjustments for age, sex, and race (30). Nevertheless, another clinical observation study in 2019 does not support the bidirectional relations between anxiety and mood disorders and change in BMI (31). Another possible reason is that our subjects were generally overweight, although obesity is a risk factor for stroke (32). The average BMI of the patients with non-PSA was 23.99 (10), making potential difficulties in detecting the BMI-based difference at a statistical significance. As for the statistical significance in the RFM in univariate analysis but not in multivariate analysis, one possible reason is that our small number of sample size resulted in low statical power. Therefore, abdominal obesity is a better evaluation index and may have a substantial ability to predict PSA.

Our study also found high fasting glucose levels were an independent risk factor for PSA. Several studies have linked blood sugar levels to obesity and obesity can cause hyperglycemia to some extent (33, 34). In the peripheral target tissues of obese patients, the number of insulin receptors is reduced and insulin is not sensitive, which can cause an increase in blood glucose (35). Previous studies have also shown a correlation between high blood sugar and anxiety (36), further confirming the correlation between obesity and PSA.

We found that both abdominal obesity and fasting blood glucose can independently predict PSA, so, is the predictive value of combining the two higher? Therefore, the ROC curve was used to further compare the predictive value between abdominal obesity, fasting blood glucose, and their integrated model, and the results showed that the combined prediction value of both groups was higher than that of the single model. It suggests that clinical doctors can predict PSA more sensitively by consciously combining abdominal obesity and fasting blood glucose, so as to intervene early.

Previous literature has shown that obesity is associated with depression (37, 38), but the correlation between obesity and PSD is rarely reported currently. In this study, our results showed that obesity parameters were not related to PSD. Therefore, further studies are needed to explore and confirm the relationship between obesity and PSD.

We assessed the association between obesity/abdominal obesity and their indicators including WC, BMI, and RFM with PSA. To the best of our knowledge, this is the first study to use abdominal obesity as an evaluation index to explore the potential association between obesity and PSA. However, our study still has the following limitations. First, patients with severe cognitive impairments and aphasia were excluded, which perhaps resulted in a certain selection bias in our study. Thus, the NIHSS scores are lower in the present population, and PSA incidence and severity are likely to be lower. Second, the short follow-up time made it impossible to assess the relationship between obesity and long-term PSA incidence and severity. Third, larger sample size may yield better results, allowing for various stratified analyses of obesity and PSA. Fourth, we did not collect data on other factors that may be associated with PSA, such as stroke lesion location, psychosocial problems, and psychosocial support. Finally, due to limited conditions in study places, we did not use a more accurate method of dual-energy X-ray absorption (DXA) to evaluate obesity (39), and this may have led to an underestimated association between obesity and PSA. We will refine these limitations in the next study.

In conclusion, we found a certain correlation between obesity-related parameters and PSA instead of PSD, suggesting that controlling obesity, especially abdominal obesity, may reduce the occurrence risk of PSA. We look forward to future studies to confirm this thesis.

## Data Availability Statement

The raw data supporting the conclusions of this article will be made available by the authors, without undue reservation.

## Author Contributions

P-LH: methodology. C-XY: validation. Y-TR: formal analysis. H-HQ: investigation. X-CH: data curation. A-YH: writing—original draft preparation. G-QH and B-LZ: writing—review and editing. J-CH and BY: supervision. All authors have read and agreed to the published version of the manuscript.

## Funding

This research was funded by grants from the Projects of National Natural Science Foundation of China, (Grant Number 81873799).

## Conflict of Interest

The authors declare that the research was conducted in the absence of any commercial or financial relationships that could be construed as a potential conflict of interest.

## Publisher's Note

All claims expressed in this article are solely those of the authors and do not necessarily represent those of their affiliated organizations, or those of the publisher, the editors and the reviewers. Any product that may be evaluated in this article, or claim that may be made by its manufacturer, is not guaranteed or endorsed by the publisher.
